# Synergistic Antibiofilm Activity between Synthetic Peptides and Ciprofloxacin against *Staphylococcus aureus*

**DOI:** 10.3390/pathogens11090995

**Published:** 2022-08-31

**Authors:** Nilton A. S. Neto, Jose T. A. Oliveira, Tawanny K. B. Aguiar, Leandro P. Bezerra, Levi A. C. Branco, Felipe P. Mesquita, Cleverson D. T. Freitas, Pedro F. N. Souza

**Affiliations:** 1 Department of Biochemistry and Molecular Biology, Federal University of Ceará, Fortaleza 60451, CE, Brazil; 2 Department of Fisheries Engineering, Federal University of Ceará, Fortaleza 60451, CE, Brazil; 3 Drug Research and Development Center, Department of Physiology and Pharmacology, Federal University of Ceará, Caixa, Fortaleza 60430, CE, Brazil

**Keywords:** synthetic peptides, biofilm, *Staphylococcus aureus*, synergism

## Abstract

*Staphylococcus aureus* is a human pathogen known to be resistant to antibiotics since the mid-20th century and is constantly associated with hospital-acquired infections. *S. aureus* forms biofilms, which are complex surface-attached communities of bacteria held together by a self-produced polymer matrix consisting of proteins, extracellular DNA, and polysaccharides. Biofilms are resistance structures responsible for increasing bacterial resistance to drugs by 1000 times more than the planktonic lifestyle. Therefore, studies have been conducted to discover novel antibacterial molecules to prevent biofilm formation and/or degrade preformed biofilms. Synthetic antimicrobial peptides (SAMPs) have appeared as promising alternative agents to overcome increasing antibiotic resistance. Here, the antibiofilm activity of eight SAMPs, in combination with the antibiotic ciprofloxacin, was investigated in vitro. Biofilm formation by *S*. *aureus* was best inhibited (76%) by the combination of *Mo*-CBP_3_-PepIII (6.2 µg mL^−1^) and ciprofloxacin (0.39 µg mL^−1^). In contrast, the highest reduction (60%) of the preformed biofilm mass was achieved with *Rc*Alb-PepII (1.56 µg mL^−1^) and ciprofloxacin (0.78 µg mL^−1^). Fluorescence microscopy analysis reinforced these results. These active peptides formed pores in the cellular membrane of *S*. *aureus*, which may be related to the enhanced ciprofloxacin’s antibacterial activity. Our findings indicated that these peptides may act with ciprofloxacin and are powerful co-adjuvant agents for the treatment of *S*. *aureus* infections.

## 1. Introduction

*Staphylococcus aureus* resistance to antibiotics keeps evolving constantly. For example, penicillin was released for use in 1941. In 1943, the first *S. aureus* isolate resistant to penicillin was discovered. In 1944, 5% of *S. aureus* isolates were resistant to penicillin, and this number increased dramatically to 80% in 1959. In addition to resistance to penicillin, *S. aureus* acquired resistance genes such as *blaZ*, *blaI*, and *blaR1*, conferring resistance to other antibiotics [1,2,3].

In 1960, methicillin, a penicillin-class semi-synthetic antibiotic, was created to combat resistant *S. aureus* by targeting beta-lactamase enzymes responsible for resistance to penicillin. Indeed, methicillin worked but not for very long. In 1961, the first report of methicillin-resistant *S. aureus* (MRSA) was documented, and the gene *mecA* was reported as responsible for the resistance. Based on the resistance to methicillin, another antibiotic, vancomycin, was employed to treat *S. aureus* infections; however, later in the 1960s emerged the vancomycin-resistant *S. aureus* (VRSA). VRSA strains possess the operon vanA, which consists of a pool of genes involved in synthesizing a modified peptidoglycan precursor not affected by vancomycin treatment. Due to the falling use of vancomycin, fluoroquinolones were used against MRSA [1,2,3,4,5]. After a few years, MRSA started to show resistance to these antibiotics, such as ciprofloxacin [1,2].

Recently, the most common antibiotics used to treat MRSA infections are lipopeptides and oxazolidinones. Even though these antibiotics are still effective, recent studies already show MRSA strains resistant to these antibiotic classes [2,4,5]. In 2017, an estimation of 119,247 *S. aureus* bloodstream infections occurred, with 19,832 associated deaths in the United States [5].

Besides the resistance to drugs, *S. aureus* can also form biofilms, which could increase resistance by 1000 times [2,4,5]. The biofilm matrix formed by *S. aureus* is composed of protein (either secreted and lysis-derived proteins), complex carbohydrates (e.g., N-acetyl-glucosamine), and extracellular DNA (eDNA) [6]. These components vary according to the strain and environmental conditions [6]. The biofilm matrix possesses a dispersal mechanism that reduces the effectiveness of drugs, increasing the resistance [7]. All the ways by which *S. aureus* acquires resistance reinforce the need to seek alternative molecules related to treating infections caused by MRSA, especially hospital-acquired infections [2,4,5].

SAMPs are an alternative treatment to the infection caused by resistant *S. aureus* [8,9,10,11]. SAMPs present many advantages since they are rationally designed based on antimicrobial proteins [9]. Some of these advantages are their mechanism of action (interaction with the cell membrane and cell wall), low allergenic potential, and high yield since they can be obtained by chemical synthesis [8,9].

Recently, our research group has shown that SAMPs derived from plant antimicrobial proteins presented great activity against *S. aureus* planktonic cells [9,10,12]. In this context, our research group evaluated the combined antibiofilm activity of those synthetic peptides with ciprofloxacin against *S. aureus* biofilm. In addition, the mechanism of action of peptides and ciprofloxacin alone or combined were evaluated by fluorescence microscopy. Altogether, the results revealed synthetic peptides enhanced the potential of ciprofloxacin against *S. aureus* in both cases inhibiting biofilm formation and reduction the mass of preformed biofilm.

## 2. Materials and Methods 

### 2.1. Biologic Material 

*S. aureus* (ATCC 25923) strain was obtained from the laboratory of toxic proteins (LabTox) from the department of biochemistry and molecular biology of the Federal University of Ceará (UFC).

### 2.2. Peptide Sequence

The synthetic peptides *Mo*-CBP_3_-PepI, *Mo*-CBP_3_-PepII, *Mo-*CBP_3_-PepIII (Oliveira et al., 2019), *Rc*Alb-PepI, *Rc*Alb-PepII, *Rc*Alb-PepIII (Dias et al., 2020), PepGAT ,and Pep KAA (Souza et al., 2020) were chemically synthesized by ChemPeptide (Shanghai, China), where they were analyzed for purity and quality (≥95%) by reverse-phase high-performance liquid chromatography and mass spectrometry.

### 2.3. Antibiofilm Assay

The assay was made in flat-bottom 96-well polystyrene microplates as described by Bezerra et al. [11]. The cell suspension was prepared using a single colony of *S. aureus* from a Petri dish with Mueller–Hinton broth (stock bacteria). The colony was transferred to Mueller–Hinton broth and incubated at 37 ℃ for 24 h. After, the cell suspension was standardized to a concentration of 10^6^ cells mL^−1^. To the assay to observe the inhibition of biofilm formation, 50 μL from the standardized cell suspension was incubated in contact with 25 μL of the peptide solution, diluted in 5% dimethyl sulfoxide (DMSO), prepared in NaCl 0.15 M (DMSO-NaCl) at different concentrations (1000 to 0.2 μg mL^−^^1^) with and 25 μL of ciprofloxacin solution, diluted in DMSO-NaCl, at different concentrations (1000 to 0.2 μg mL^−1^) in the dark, at 37 ℃, for 48 h. The combination of peptide and antibiotic was made from the highest concentration of the peptide combined with the lowest antibiotic concentration and vice versa. 

After the incubation period, the supernatant was removed carefully from each well and washed one time with sterile NaCl 0.15 M solution. Then, the biofilm was fixated with 100 μL of 99% methanol for 15 min, and after drying, the biofilm was stained with 200 μL of 0.1% violet crystal solution. Then, the wells were washed three times with sterile 0.15 M NaCl solution, and the remaining crystal was solubilized with 200 μL of 33% acetic acid (v/v) solution. The absorbances were obtained via a microplate reader (BioTek^TM^ Epoch, BioTek Instruments, Inc., Winooski, VT, USA) at 600 nm. The experiment was repeated three times.

The assay to observe the preformed biofilm reduction was conducted the same as described above but with some adjustments. Initially, 50 μL of the standardized cell suspension were incubated in flat-bottom, 96-well microplates in the dark, at 37 ℃, for 24 h, to biofilm formation. The preformed biofilm was incubated with 25 μL of the peptides or ciprofloxacin alone or in combination as described above. The microplates were incubated in the dark, at 37 ℃, for 24 h. After incubation, the biofilm was washed, dried, and stained, and absorbance was taken as above. 

### 2.4. Biofilm Integrity Determined by Fluorescence Microscopy

The assay was conducted as described by Bezerra et al. [11]. Biofilm was produced, and the assay was conducted as described in the antibiofilm assay. Still, instead of using 96-well microplates, the assay was made in coverslips inside 6-well microplates. After incubation, the coverslips were washed three times with sterile 0.15 M NaCl. Afterward, the coverslips were incubated with an aqueous solution of propidium iodide (PI, 10 μM) in the dark, at 37 ℃, for 30 min. Then, they were washed with sterile 0.15 M NaCl three times to remove the excess PI. Then, the coverslips were observed with a fluorescence microscope (Olympus System Bx 60, Tokyo, Japan) in a 535 nm excitation and 617 nm wavelength.

### 2.5. Overproduction of Reactive Oxygen Species (ROS)

The ROS overproduction was determined following the method described by Bezerra et al. [13]. The assays were conducted as the same for PI analysis. Then, 20 µL of 2′,7′ dichlorohfluorescein diacetate (DCFH-DA, Sigma, St. Louis, MI, USA) was added and incubated in the dark for 30 min at 24 °C. Finally, the biofilms were washed with 0.15 M NaCl and observed under a fluorescence microscope (Olympus System BX 41, Tokyo, Japan) with an excitation wavelength of 488 nm and an emission wavelength of 525 nm.

### 2.6. Hemolytic Assay 

The hemolytic activities against A, B, and O types of human erythrocytes of peptides and ciprofloxacin alone or in combination were done following the methodology by Bezerra et al. [13]. The concentrations of all solutions were the same as used in the synergism assays. The blood types were provided by the Hematology and Hemotherapy Center of Ceará (Brazil). The blood was collected in a tube with heparin (5 IU mL^−1^, Sigma Aldrich, São Paulo, Brazil), centrifuged at 300× *g* for 5 min at 4 °C, washed with sterile 0.15 M NaCl, and diluted to a concentration of 2.5%. Each blood type was incubated (100 µL) with peptides and ciprofloxacin alone or in combination for 30 min at 37 °C and then centrifuged 300× *g* for 5 min at 4 °C (centrifuge Eppendorf 5810, Hannover, Germany). Supernatants were collected and the absorbance read at 414 nm. DMSO-NaCl solution (0%) and 0.1% Triton X-100 (100%) were used as negative and positive controls for hemolysis, respectively. The followed equation calculated the hemolysis: [(Abs_414nm_ of the sample treated with peptides or drugs-Abs_414nm_ of samples treated with DMSO-NaCl)/[(Abs_414nm_ of samples treated with 0.1% TritonX-100-Abs_414nm_ of samples treated with DMSO-NaCl] × 100.

### 2.7. Statistical Analysis

All assays were performed in triplicate, and the values were expressed as the mean ± standard error. All quantitative data were submitted to one-way ANOVA followed by the Tukey test using GraphPad Prism 6.01 (GraphPad Software Company, Santa Clara, CA, USA), with a *p* < 0.05. 

## 3. Results

### 3.1. Combined Antibiofilm Activity of Synthetic Peptides and Ciprofloxacin against S. aureus 

**Figure 1:**
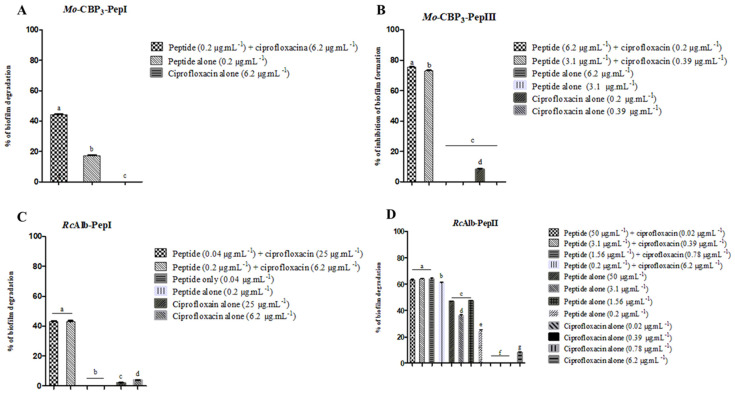
Combined antibiofilm activity of synthetic peptides and ciprofloxacin against *S. aureus* biofilm. (**A**) Mo-CBP3-PepI, (**B**) Mo-CBP3-PepIII, (**C**) RcAlb-PepI, and (**D**) RcAlb-PepII. Different lowercase letters indicate statically significant difference compared to DMSO-NaCl by analysis of variance (*p* < 0.05).

Synthetic peptides and ciprofloxacin presented different behaviors toward *S. aureus* biofilm. Ciprofloxacin alone could not successfully inhibit the formation or reduce the mass of preformed biofilms. In the case of synthetic peptides, the activity alone was very low but still present. However, combinations between some synthetic peptides and ciprofloxacin presented promising results (Figure 1). Out of eight synthetic peptides, four presented great results in combination with ciprofloxacin *Mo*-CBP3-PepI, *Mo*-CBP3-PepIII, *Rc*Alb-PepI, and *Rc*Alb-PepII. All the effective combinations are presented in Figure 1.

Although twelve combinations of *Mo*-CBP3-PepI and ciprofloxacin were tested, only one was effective in reducing the biomass of *S. aureus* preformed biofilm (Figure 1A). In contrast, *Mo*-CBP3-PepI (0.2 µg mL^−1^) and ciprofloxacin (6.2 µg mL^−1^) alone reduced, respectively, 19 and 0% of preformed biofilm from *S. aureus*, and combined, the reduction of preformed biofilm increased to 50% (Figure 1A). The combination between *Mo*-CBP3-PepIII and ciprofloxacin had the best efficacy in the inhibition of biofilm formation (Figure 1B). Alone, at concentrations of 6.2 µg mL^−1^ and 3.1 µg mL^−1^, *Mo*-CBP3-PepIII did not inhibit the biofilm formation of *S. aureus* to any extent. Ciprofloxacin alone, at concentrations of 0.2 µg mL^−1^ and 0.39 µg mL^−1^, only inhibited the biofilm formation by 10% at 0.2 µg mL^−1^ (Figure 1B). Two combinations of *Mo*-CBP3-PepIII and ciprofloxacin showed the best results. *Mo*-CBP3-PepIII (6.2 µg mL^−1^) and ciprofloxacin (0.39 µg mL^−1^) and *Mo*-CBP3-PepIII (3.1 µg mL^−1^) and ciprofloxacin (0.2 µg mL^−1^) inhibited the biofilm formation, respectively, by 73 and 76% (Figure 1B). 

*Rc*Alb-PepI worked in two combinations with ciprofloxacin to reduce the mass of preformed biofilm (Figure 1C). Two combinations made of *Rc*Alb-PepI (0.04 µg mL^−1^) and ciprofloxacin (25 µg mL^−1^) and *Rc*Alb-PepI (0.2 µg mL^−1^) and ciprofloxacin (6.2 µg mL^−1^) reduced 40% of the preformed biofilm of *S. aureus* (Figure 1C). Interestingly, *Rc*Alb-PepI and ciprofloxacin alone in both concentrations were not effective to reduce the biomass of biofilm from *S. aureus*. The combinations made by *Rc*Alb-PepI (50 µg mL^−1^) and ciprofloxacin (0.02 µg mL^−1^), *Rc*Alb-PepI (3.1 µg mL^−1^) and ciprofloxacin (0.39 µg mL^−1^), *Rc*Alb-PepI (1.56 µg mL^−1^) and ciprofloxacin (0.78 µg mL^−1^), and *Rc*Alb-PepI (0.2 µg mL^−1^) and ciprofloxacin (6.2 µg mL^−1^) reduced around 65% of *S. aureus* biofilm biomass (Figure 1D). At these concentrations alone, ciprofloxacin was not effective in reducing the biomass of *S. aureus* preformed biofilm. 

### 3.2. Action Mechanisms of Synthetic Peptides

#### 3.2.1. Membrane Pore Formation by Propidium Iodide Uptake

The mechanisms of action behind the activity of synthetic peptides and ciprofloxacin, either alone or in combination, were evaluated by fluorescence microscopy. The best activities shown in Figure 1 were analyzed by fluorescence microscopy to observe the pore formation on the membrane of cells by the PI uptake. The combinations were as follows: *Mo*-CBP3-PepI (0.2 µg mL^−1^) and ciprofloxacin (6.2 µg mL^−1^) against preformed biofilm (Figure 1A), *Mo*-CBP3-PepIII (6.2 µg mL^−1^) and ciprofloxacin (0.2 µg mL^−1^) against the formation of biofilm (Figure 1B), *Rc*Alb-PepI (0.04 µg mL^−1^) and ciprofloxacin (25 µg mL^−1^) against preformed biofilm, and *Rc*Alb-PepII (50 µg mL^−1^) and ciprofloxacin (0.02 µg mL^−1^) against preformed biofilm (Figure 1D). 

**Figure 2:**
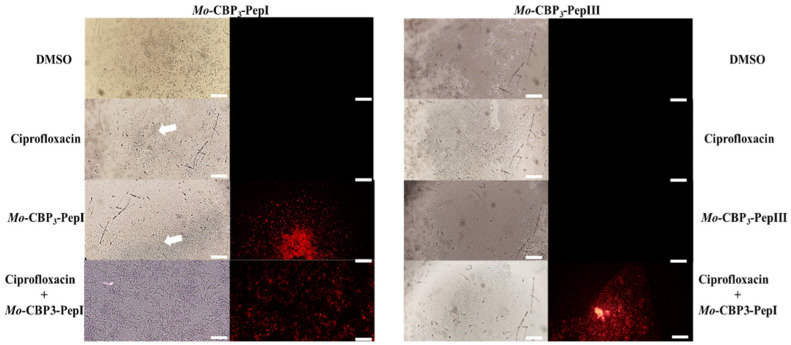
Fluoresce microscopy shows the effects of the *Mo*-CBP3-PepI and *Mo*-CBP3-PepIII + ciprofloxacin on the membrane of biofilm cells *S. aureus*. DMSO: Control treatment with DMSO-NaCl solution. Ciprofloxacin: at 6.2 µg mL^−1^ (left panel) and 0.2 µg mL^−1^ (right panel). *Mo*-CBP3-PepI alone at 0.2 µg mL^−1^; *Mo*-CBP3-PepIII alone at 6.2 µg mL^−1^; *Mo*-CBP3-PepI and ciprofloxacin (0.2 µg mL^−1^ and 6.2 µg mL^−1^, respectively); *Mo*-CBP3-PepIII and ciprofloxacin (6.2 µg mL^−1^ and 0.2 µg mL^−1^, respectively). In the left panel is the assay of reduction of preformed biofilm, and in the right panel is the inhibition of biofilm formation. Bars = 100 µm.

**Figure 3:**
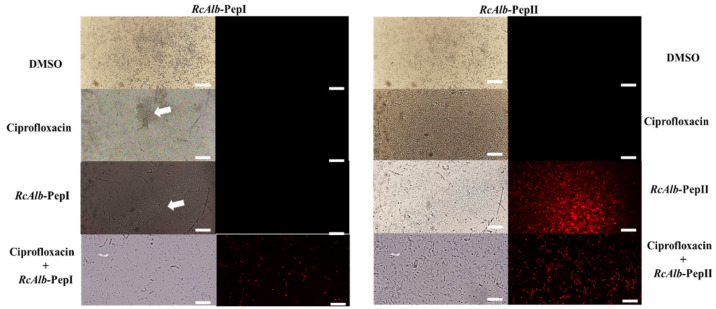
Fluoresce microscopy shows the effects of the *Rc*Alb-PepI and *Rc*Alb-PepIII and ciprofloxacin on the membrane of biofilm cells *S. aureus*. DMSO: Control treatment with DMSO-NaCl solution. Ciprofloxacin: at 25 µg mL^−1^ (left panel) and 0.02 µg mL^−1^ (right panel). *Rc*AlbPepI alone at 0.04 µg mL^−1^; *Rc*Alb-PepIII alone at 50 µg mL^−1^; *Rc*Alb-PepI and ciprofloxacin (0.04 µg mL^−1^ and 25 µg mL^−1^, respectively); *Rc*AlbPepIII and ciprofloxacin (50 µg mL^−1^ and 0.02 µg mL^−1^, respectively). Both panels show reduction of biofilm mass assay. Bars = 100 µm.

PI is a dye that interacts with nuclei acids by releasing red fluorescence. However, PI can only permeate through a damaged membrane; a healthy membrane does not allow the movement of PI by it. The treatment presenting red fluorescence indicates damage to cell membranes. As expected, DMSO-NaCl and ciprofloxacin did not induce damage to the cell membrane given the absence of red fluorescence (Figure 2 and Figure 3). The mechanism of action of ciprofloxacin does not involve damage to the membrane, which was confirmed by PI assay uptake. The *Mo*-CBP3-PepI (0.2 µg mL^−1^) alone was able to induce pore formation in *S. aureus* biofilm cells, and fluorescence was even higher in the combination of *Mo*-CBP3-PepI (0.2 µg mL^−1^) and ciprofloxacin (6.2 µg mL^−1^) (Figure 2).

In contrast, *Mo*-CBP3-PepIII (6.2 µg mL^−1^) alone was not able to induce pore formation of biofilm cells of *S. aureus*, as no fluorescence was detected (Figure 2). However, the combination made by *Mo*-CBP3-PepIII (6.2 µg mL^−1^) and ciprofloxacin (0.2 µg mL^−1^) induced the releasing of red fluorescence, suggesting both *Mo*-CBP3-PepIII and ciprofloxacin work together to induce pore formation of biofilm cells of *S. aureus* (Figure 2). 

In the case of *Rc*Alb-PepI, only in combination with *Rc*Alb-PepI (0.04 µg mL^−1^) and ciprofloxacin (25 µg mL^−1^) was it possible to induce pore formation on the membrane of *S. aureus* in biofilm (Figure 3). Alone, DMSO, ciprofloxacin (alone), and *Rc*Alb-PepI (at 6.2 µg mL^−1^ alone) did not induce pore formation on the membrane of *S. aureus* in biofilm (Figure 3). In contrast, *Rc*Alb-PepII alone (at 50 µg mL^−1^) or in combination and *Rc*Alb-PepII and ciprofloxacin (50 µg mL^−1^ and 0.02 µg mL^−1^, respectively) were able to induce pore formation on the membrane as revealed by PI uptake. DMSO and ciprofloxacin could not induce pore formation (Figure 3).

#### 3.2.2. ROS Overproduction

**Figure 4:**
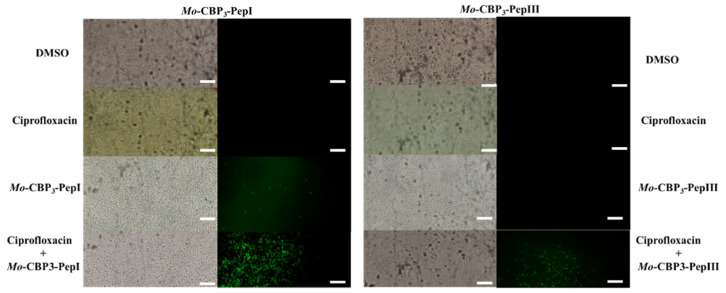
Fluoresce microscopy showed ROS overproduction induced by *Mo*-CBP3-PepI and *Mo*-CBP3-PepIII and ciprofloxacin ROS overproduction in *S. aureus* biofilm. DMSO: Control treatment with DMSO-NaCl solution. Ciprofloxacin: at 6.2 µg mL^−1^ (left panel) and 0.2 µg mL^−1^ (right panel). *Mo*-CBP3-PepI alone at 0.2 µg mL^−1^; *Mo*-CBP3-PepIII alone at 6.2 µg mL^−1^; *Mo*-CBP3-PepI and ciprofloxacin (0.2 µg mL^−1^ and 6.2 µg mL^−1^, respectively); *Mo*-CBP3-PepIII + ciprofloxacin (6.2 µg mL^−1^ and 0.2 µg mL^−1^, respectively). In the left panel is the assay of reduction of preformed biofilm, and in the right panel is the inhibition of biofilm formation. Bars = 100 µm.

**Figure 5:**
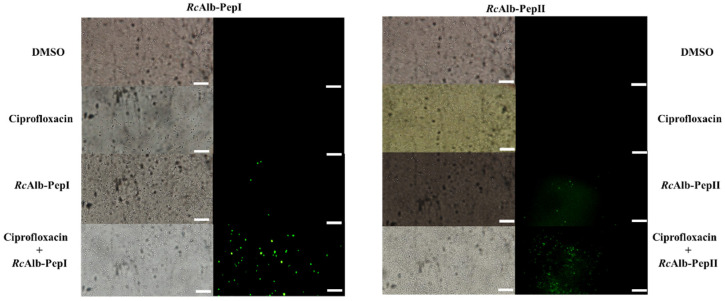
Fluoresce microscopy showed ROS overproduction induced by *Rc*Alb-PepI, *Rc*Alb-PepIII, and ciprofloxacin *S. aureus* biofilm. DMSO: Control treatment with DMSO-NaCl solution. Ciprofloxacin: at 25 µg mL^−1^ (left panel) and 0.02 µg mL^−1^ (right panel). *Rc*Alb-PepI alone at 0.04 µg mL^−1^; *Rc*Alb-PepIII alone at 50 µg mL^−1^; *Rc*Alb-PepI and ciprofloxacin (0.04 µg mL^−1^ and 25 µg mL^−1^, respectively); *Rc*Alb-PepIII and ciprofloxacin (50 µg mL^−1^ and 0.02 µg mL^−1^, respectively). Both panels are reduction of biofilm mass assay. Bars = 100 µm.

The evaluation of ROS overproduction revealed that DMSO-NaCl and ciprofloxacin did not induce ROS in any treatments (Figure 4 and Figure 5). *Mo*-CBP3-PepI slightly induced the ROS production in biofilm cells of *S. aureus* (Figure 4). This ROS production was even higher in the combination of *Mo*-CBP3-PepI with ciprofloxacin (Figure 4). In contrast, *Mo*-CBP3-PepIII only induced ROS overproduction in biofilm cells of *S. aureus* when combined with ciprofloxacin (Figure 4). Alone, *Mo*-CBP3-PepIII, DMSO, and ciprofloxacin did not induce any ROS accumulation.

Interestingly, both *Rc*Alb-PepI and *Rc*Alb-PepII induced ROS accumulation alone or in combination with ciprofloxacin. Both peptides presented similar behavior. Alone, they induced less ROS accumulation in biofilm cells of *S. aureus* than combined with ciprofloxacin (Figure 5). In both cases, the combination with ciprofloxacin was effective in ROS accumulation. 

### 3.3. Hemolytic Action

As reported in many other studies from our research group, synthetic peptides are not toxic to human red blood cells (HRBC) [10,12]. Additionally, it has been shown that during synergistic action, peptides, besides enhancing drugs’ effects, also reduce their toxicity to HRBC [11,13].

Here, the hemolytic potential of peptides and ciprofloxacin were assayed alone or in combination (Table 1). Positive control for hemolysis, 0.1% Triton X-100, induced 100% hemolysis in all three types of HRBC (Table 1). Ciprofloxacin in the highest concentration tested (1000 µg mL^−1^) hemolyzed 100% of all types of HRBC (Table 1). One of the concentrations of ciprofloxacin (25 µg mL^−1^) used in combination with peptides, when alone, still induced 45, 51, and 34% of hemolysis, respectively, in type-A, -B, and -O of HRBC (Table 1). The other concentrations of ciprofloxacin were not toxic to HRBC. As expected, even at higher concentrations, peptides were not toxic to HRBC (Table 1).

An interesting result was found during the hemolysis assay with combinations. All combinations of peptides and ciprofloxacin were not toxic to HRBC (Table 1). Even ciprofloxacin at 25 µg mL^−1^, which was toxic alone, did not present as toxic to HRBC when combined with *Rc*Alb-PepI (Table 1).

## 4. Discussion

There is no doubt that *S. aureus* is a major health issue due to the severity of hospital-acquired infections caused by *S. aureus*, which is worsened by its resistance to many antibiotics available nowadays. With the biofilm formation, the gravity of the situation is even more complicated because the biofilm is a well-evolved resistance structure formed to avoid potential threats. *S. aureus* biofilms cause concern due to their ability to easily form biofilms on many different surfaces [3,4,5,14,15,16].

As described above, *S. aureus* quickly accumulates mutations, resulting in resistance to many drugs. Those are antibiotics from different groups with different modes of action, reinforcing the ability of *S. aureus* to acquire resistance [15,17]. This high acquisition of mutation to many drugs results from a genetic variation associated with phenotypic plasticity presented by *S. aureus* in response to environmental insults, which allows *S. aureus* to adapt to environmental changes to maintain growth, reproduction, and infection process [18]. For example, Gardete and Tomasz [19] analyzed that *S. aureus* cells cultivated in a media supplied with vancomycin presented a thickened cell wall compared to cells cultivated in a vancomycin-free medium. 

Based on that, developing new molecules or even an association of molecules could be a hopeful strategy to cope with *S. aureus* resistance. At this point, synthetic peptides could be a great alternative to be used alone to develop new drugs or even act as an adjuvant to improve drugs already used. 

The synthetic peptides used in this work have already shown antimicrobial activity against many human pathogens, such as dermatophyte fungi, pathogenic yeasts, SARS-CoV-2, and bacteria such as *S. aureus* itself [10,12,20,21,22]. The hypothesis behind this work was whether these peptides could inhibit the formation or even reduce *S. aureus* biofilm biomass alone or in combination with ciprofloxacin. 

Many studies have been carried out concerning the synergistic effect of antimicrobial peptides in combination with commercial drugs. Bessa et al. [23] and Martinez et al. [24] presented antimicrobial peptides with antibiofilm activity against resistant *P. aeruginosa* strains and synergistic effects in combination with antibiotics such as meropenem and ciprofloxacin. However, the mechanisms wherewith this synergism happens are not well explained yet. 

One of the proposed models most accepted by the scientific community is that the vast majority of antimicrobial peptides alter the membrane permeability, making it possible for such drugs to enter cells and allow them to interact with their targets [25]. Our findings show an increase in inhibition and degradation of biofilms when ciprofloxacin is combined with peptides, even at very low concentrations. For example, ciprofloxacin at 25 μg mL^−1^ alone reduced only 8% of *S. aureus* preformed biofilm. However, the combination of ciprofloxacin at 25 μg mL^−1^ with *Rc*Alb-PepI at 0.04 μg mL^−1^ increased this reduction up to 45% (Figure 1). *Rc*Alb-PepI at 0.04 μg mL^−1^ alone was able to induce pore formation in *S. aureus* biofilm cells, which could have facilitated the movement of ciprofloxacin to the cell cytoplasm, improving its action. It is essential to notice that our peptides increase the action of ciprofloxacin at very low concentrations (Table 1). While Bessa et al. [23] showed synergistic concentrations at 8, 16, 32, and 128 μg mL^−1^, our peptides presented a synergistic effect at concentrations ten to hundreds of times lower than those.

Ciprofloxacin is an antibiotic classified in the fluoroquinolone group. Its mechanism of action relies on the inhibition of DNA replication by interacting and inhibiting the bacterial DNA topoisomerase IV enzyme [26]. Ciprofloxacin has been employed to treat abdominal infections, diarrhea, respiratory tract infections, and skin infections [26,27,28,29,30,31]. By attacking an intracellular protein, ciprofloxacin has to be transported to the cytoplasm, passing by the membrane using porin transmembrane proteins as a channel [28]. The typical resistance mechanisms of *S. aureus* alter the cytoplasmic concentration of ciprofloxacin, such as increasing the concentration efflux pumps and developing mutations in the gene to produce a new or modified topoisomerase IV [32]. In this sense, combining ciprofloxacin with molecules that increase cytoplasmic concentration could be an excellent alternative to overcome the resistance of *S. aureus*.

Here, we showed that synthetic peptides *Mo*-CBP3-PepI, *Rc*Alb-PepI, and *Rc*Alb-PepI alone induced pore formation in biofilm cells of *S. aureus* (Figure 2 and Figure 3). PI in FM analysis helped us understand how mechanism peptides improve ciprofloxacin actions. Membrane pores induced by peptides allow the movement of PI, which has a molecular weight of 668.39 Da, by the membrane. Thus, the movement of PI by the membrane indicates at least the presence of a pore of that size (Figure 2 and Figure 3). Ciprofloxacin has a molecular weight of 331.34 Da. Based on that, it is feasible to suggest that the pores induced by peptides facilitate the movement of ciprofloxacin by the membrane, increasing its concentration in the cytoplasm and thus the activity. Recently, it has been shown that *Mo*-CBP3-PepI, *Rc*Alb-PepI, and *Rc*Alb-PepI can also induce pores with a size of 6 kDa in the membrane of several pathogens [10,33]. This information strengthens our hypothesis about how peptides enhance the action of ciprofloxacin against *S. aureus*.

Here, the second set of FM experiments revealed that all the combinations between peptides and ciprofloxacin lead to an overaccumulation of ROS biofilm cells of *S. aureus* (Figure 4 and Figure 5). Only ciprofloxacin and *Mo*-CBP3-PepIII alone were not able to induce ROS accumulation. ROS, essentially H_2_O_2_, is vital to the signaling process that leads to biofilm formation at earlier and later stages such as development and maturation [34]. However, cells need to strictly regulate the levels of ROS because from a beneficial to a lethal effect is a fine line easy to cross. Any imbalance in ROS levels leading to high accumulation is lethal because it destroys molecules essential to cell life, such as carbohydrates, nucleic acids, proteins, and lipids, triggering programmed cell death [35]. In addition, to facilitate the entry of ciprofloxacin into the cytoplasm, peptides induce additional stress via ROS accumulation on cells in biofilms of *S. aureus*, which makes it difficult for *S. aureus* to fight back against the combined action of peptides and ciprofloxacin.

Ciprofloxacin is known to cause a large number of collateral effects in patients during treatment [26,27,28,29,30,31,36]. Collateral effects caused by ciprofloxacin go from mild, such as nausea and diarrhea, to severe, such as seizures, neuropathy, photosensitivity, and hyper or hypoglycemia [26,27,28,29,30,31,36]. Additionally, it has been reported that treatment with ciprofloxacin induced interstitial nephritis and autoimmune hemolytic anemia [36]. 

Here, our data (Table 1) revealed that in addition to enhancing the action of ciprofloxacin, peptides reduced their hemolytic activity against HRBC. Alone, the ciprofloxacin at 25 µg mL^−1^ alone induced high levels of HRBC. However, the same concentration of ciprofloxacin in combination with peptide RcAlb-PepI at 0.04 µg mL^−1^ did not present any toxicity to HRBC. This is an exciting result because it reinforces the potential of peptides as adjuvants in drug formulations to treat infections caused by *S. aureus*. As revealed in other studies, the peptides used in this study were not toxic to human cells, and zebrafish embryos strengthen the clinical application of peptides [10,12,22].

## 5. Conclusions

Here, we presented four synthetic peptides that enhanced the activity of ciprofloxacin against biofilms of *S. aureus*. The mechanism of the combined effect is possible by increasing the cytoplasmatic concentration of ciprofloxacin supported by pores on the membrane of *S. aureus* cells. Additionally, peptides reduced the toxicity of ciprofloxacin to HRBC. Considering all these findings, it is possible to suggest that the peptides studied are a considerable option to surpass the resistance of *S. aureus* strains to antibiotics such as ciprofloxacin.

## Figures and Tables

**Table 1:** Hemolytic activity of synthetic peptides, antifungal drugs, and their combination toward human red blood cells.

Peptides/Combinations	% Hemolysis		
	Type-A Blood	Type-B Blood	Type-O Blood
0.1% Triton X-100	100 ± 0.001	100 ± 0.001	100 ± 0.005
DMSO-NaCl Solution	0	0	0
Ciprofloxacin (1000 µg mL^−1^)	100 ± 0.007	100 ± 0.003	100 ± 0.005
Ciprofloxacin (25 µg mL^−1^)	45 ± 0.004	51 ± 0.002	34 ± 0.006
Ciprofloxacin (0.2 µg mL^−1^)	0	0	0
Ciprofloxacin (0.02 µg mL^−1^)	0	0	0
*Mo*-CBP_3_-PepI (1000 µg mL^−1^)	0	0	0
*Mo*-CBP_3_-PepIII (1000 µg mL^−1^)	0	0	0
RcAlb-PepI (1000 µg mL^−1^)	0	0	0
RcAlb-PepII (1000 µg mL^−1^)	0	0	0
*Mo*-CBP_3_-PepI (0.2 µg mL^−1^) and ciprofloxacin (6.2 µg mL^−1^)	0	0	0
*Mo*-CBP_3_-PepIII (6.2 µg mL^−1^) and ciprofloxacin (0.2 µg mL^−1^)	0	0	0
*Rc*Alb-PepI (0.04 µg mL^−1^) and ciprofloxacin (25 µg mL^−1^)	0	0	0
*Rc*Alb-PepII (50 µg mL^−1^) and ciprofloxacin (0.02 µg mL^−1^)	0	0	0

## Data Availability

The data supporting this study’s findings are available on request from the corresponding author.
